# Anatomical evidence links the stomach to the central amygdala, a region responsive to local GLP-1R agonist induced feeding and nausea-like behaviors in male mice

**DOI:** 10.3389/fendo.2026.1740052

**Published:** 2026-01-28

**Authors:** Hui Yang, Wenxiang Yu, Yunling Gao, Jie Wang, Shaoyong Xu

**Affiliations:** 1 Institute of Neuroscience and Brain Diseases, Xiangyang Central Hospital, Affiliated Hospital of Hubei University of Arts and Science, Xiangyang, Hubei, China; 2 Department of Endocrinology, Xiangyang Central Hospital, Affiliated Hospital of Hubei University of Arts and Science, Xiangyang, Hubei, China; 3 Songjiang Hospital and Songjiang Research Institute, Shanghai Key Laboratory of Emotions and Affective Disorders, Shanghai Jiao Tong University School of Medicine, Shanghai, China

**Keywords:** CeA, liraglutide, feeding, nausea-like behaviors, central regulation

## Abstract

**Background:**

The glucagon-like peptide-1 receptor (GLP-1R) agonist liraglutide is an effective therapeutic agent for obesity, primarily through its ability to suppress appetite and delay gastric emptying. However, the central neural substrates mediating its effects on food intake remain incompletely defined.

**Methods:**

Male mice received subcutaneous liraglutide injections in the cervical region to evalutates its effects on feeding behavior and body weight regulation. Retrograde transsynaptic tracing using pseudorabies virus (PRV) was employed to identify central amygdala (CeA) involvement in gastric-related neural circuits. The functional role of the CeA in feeding regulation was examined using chemogenetic and optogenetic activation, while local microinjection of GLP-1R agonists or antagonists into the CeA was used to evaluate receptor-specific effects.

**Results:**

Gastric wall injection of PRV anatomically revealed a direct connection between the stomach and the CeA. Site-specific administration of GLP-1R agonists into the CeA induced hypophagia and nausea-like behaviors in male mice.

**Conclusions:**

This study provides anatomical evidence that the CeA of male mice is involved in gastric regulatory circuits, and shows that the CeA responds to site-specific GLP-1R activation to induce hypophagia and nausea-like behaviors.

## 1 Introduction

Obesity is a chronic and progressive condition that is closely associated with multiple comorbidities, placing a substantial burden on individuals, healthcare systems, and socioeconomic development (1). Globally, more than one billion people are affected by obesity, underscoring the urgent need for effective therapeutic strategies (2). Glucagon-like peptide-1 receptor agonists (GLP-1RAs), including liraglutide and semaglutide, have emerged as promising pharmacological interventions for obesity management (3). Liraglutide has been shown to reduce appetite and delay gastric emptying, thereby promoting weight loss in obese individuals (4). However, the central neural mechanisms underlying liraglutide treatment for obesity remain incompletely understood.

Current research on central glucagon-like peptide-1 receptor (GLP-1R) signaling has primarily focused on the hypothalamus and hindbrain. In the arcuate nucleus (ARC), pro-opiomelanocortin/cocaine- and amphetamine-regulated transcript neurons expressing GLP-1Rs can directly bind peripherally administered liraglutide, leading to sustained neuronal depolarization (5). However, anorectic responses to systemic GLP-1RA treatment persist even after genetic deletion of hypothalamic GLP-1Rs (6). Previous study has shown that inhibition of vagal afferent signaling effectively reduces exendin-4 (Ex-4)-induced c-Fos expression in the paraventricular hypothalamus (PVH), but increases the number of c-Fos-expressing cells in the amygdala, lateral external parabrachial nucleus, caudal ventrolateral medulla, and dorsal vagal complex (DVC) (7). Subsequently, cell-specific ablation studies targeting GLP-1Rs in the hindbrain DVC, including the dorsal motor nucleus of the vagus, area postrema, and nucleus tractus solitarius (NTS), demonstrate that DVC GLP-1Rs are critical for the anorectic effects of peripherally administered exendin-4 and semaglutide (8). However, the precise circuit-level mechanisms and compensatory pathways underlying these effects remain incompletely understood.

The amygdala, a key limbic structure composed of the central, basolateral, and lateral nuclei, plays essential roles in emotional processing, reward, and motivated behaviors (9). Beyond its established roles in fear and stress responses, increasing evidence indicates that the central amygdala (CeA) is also involved in regulating feeding behavior associated with aversion or reward (10). Lesions of the CeA attenuate the anorectic effects of GLP-1R agonists, particularly in the context of palatable food intake (11). Notably, GLP-1R is broadly expressed in the CeA, with enrichment in specific subregions and partial overlap with protein kinase C delta (PKCδ)-expressing neurons (12). PKCδ^CeA^ neurons, primarily located in the lateral and capsular subdivisions (13), have been implicated in appetite suppression and meal termination through inputs from calcitonin gene-related peptide-expressing neurons in the NTS and parabrachial nucleus (9, 14). GLP-1R activation has been shown to engage PKCδ-related intracellular signaling pathways in other brain regions (15), suggesting a potential functional convergence between GLP-1R signaling and CeA feeding circuits. In addition, nausea induced by intraperitoneal administration of cisplatin markedly increases the mRNA expression of AMPA and NMDA glutamate receptor subunits within the CeA (16). Similarly, Cai et al. reported that LiCl-induced anorexia and nausea-like behaviors robustly activate PKCδ-expressing neurons in the central lateral amygdala (17). Meanwhile, restraint stress models activate GABAergic neurons in the CeA while concurrently suppressing neurons in the DVC, resulting in delayed gastric emptying and reduced gastric motility (18). He et al. further demonstrated that chemogenetic activation of GABAergic neurons in the CeA-lateral hypothalamus pathway leads to gastrointestinal dysmotility in mice (19). Collectively, these studies indicate that nausea-like behaviors are associated with activation of the CeA, whereas activation of CeA GABAergic neurons, in turn, induces gastric dysfunction. Taken together, these findings strongly suggest the existence of functional feedback between the CeA and the stomach. However, definitive anatomical evidence is still lacking, and whether stereotaxic delivery of GLP-1R agonists into the CeA similarly elicits nausea-like behaviors remains to be conclusively determined.

In this study, we found that tail vein injection of liraglutide robustly activated the CeA Fos induction. Retrograde transsynaptic pseudorabies virus (PRV) injection into the gastric wall directly revealed an anatomical connection between the stomach and the CeA. Moreover, the CeA was able to modulate feeding behavior and nausea-like behaviors in male mice through chemogenetic and optogenetic approaches, as well as via stereotaxic microinjection of GLP-1R agonists.

## 2 Materials and methods

### 2.1 Animals

The experimental protocol was approved by the Ethics Committee of Xiangyang Central Hospital (Protocol No. 2025-183) and were conducted in accordance with the Animal Research: Reporting of *In Vivo* Experiments guidelines and the Guide for the Care and Use of Laboratory Animals. All male C57BL/6J mice (RRID: MGI:5650797) were purchased from Hunan SJA Laboratory Animal Company. Animals were housed five per cage under a 12:12-h light–dark cycle at 24**–**26 °C with 45**–**65% humidity (20). The mice had access to standard chow and water *ad libitum*, except during fasting experiments and kaolin diet treatment (21). Mice were group-housed except during measurements of daily food intake, when they were singly housed (22). Mice from the same litters were assigned to different treatment groups. Healthy mice aged 6 to 8 weeks were used for surgical procedures.

### 2.2 Subcutaneous injection of liraglutide into the neck

Mice in the test group received daily subcutaneous injections of liraglutide (400 µg/kg/d; Novo Nordisk A/S, Bagsværd, Denmark) into the dorsal neck region at 8:00 a.m. Liraglutide was dissolved in 0.9% saline (NaCl, Sinopharm Chemical Reagent Co., Ltd). Control mice received equivalent volumes of saline. At 24 h post-injection, food and water intake were measured and body weights were recorded. This regimen was maintained for seven consecutive days. On day 8, tail vein blood samples were collected for measurement of blood glucose levels using a glucometer (Shanghai Roche Pharmaceuticals Co., Ltd.).

### 2.3 Injecting into the gastric wall with PRV

After induction of anesthesia (1% pentobarbital sodium dissolved in 0.9% saline, 50 mg/kg body weight, intraperitoneal injection) and confirmation of loss of consciousness, mice were placed in a supine position on the surgical table. The abdominal skin was shaved and disinfected under aseptic conditions. A midline laparotomy incision (0.8-1.5 cm) was made to expose the stomach. Three injection sites were identified along the greater curvature (superior, mid-body, and inferior regions). Each site received a 500 nL microinjection of a retrograde transsynaptic PRV (PRV-CAG-EGFP; titer: 5.0 × 10^9^ vg/ml; Braincase, China) (23, 24).

After a 5-day recovery period, mice were transcardially perfused with 20 mL of 0.9% saline followed by 20 mL of 4% paraformaldehyde (PFA) (4% PFA was dissolved in 0.9% saline.). Brains were then harvested, post-fixed in 4% PFA for 6 h, and subsequently cryoprotected in 20% and 30% sucrose solutions (Sucrose solutions were dissolved in 0.9% saline.). After post-fixation and dehydration, brains were coronally sectioned at a thickness of 40 µm using a cryostat microtome (Thermo Fisher Scientific, Waltham, MA, USA). According to *The Mouse Brain in Stereotaxic Coordinates*, 4th edition (Paxinos & Franklin, 2013), the PVH spans an anteroposterior bregma range from −0.58 to −1.22 mm, corresponding to a total length of approximately 0.64 mm. With a section thickness of 40 μm, this region yields 16 coronal sections. Similarly, the CeA extends along the anteroposterior axis from −1.22 to −1.94 mm, with a total length of approximately 0.72 mm, corresponding to 18 coronal sections at a thickness of 40 μm. Free-floating sections were collected in phosphate-buffered saline (PBS) (8 g/L NaCl, 0.2 g/L KCl, 1.44 g/L Na_2_HPO_4_, and 0.24 g/L KH_2_PO_4_ were dissolved in 1 L ddH_2_O.), mounted onto glass microscope slides, and cover-slipped. Whole-slide imaging was performed using an Olympus VS120 slide scanner (Olympus Corporation, Tokyo, Japan).

### 2.4 Tail vein injection of liraglutide

Following restraint of the mice tail, polyethylene capillary tubing was connected to a sterile insulin needle and the system was primed to expel air bubbles prior to venipuncture. After confirming venous access by observing blood flashback into the tubing, liraglutide (100 μg/kg) was administered at a rate of 0.15 mL/min. Control mice received equivalent volumes of saline. Following injection, mice were allowed to move freely for 30 minutes before brain tissues were collected as described in Section 2.3.

### 2.5 Immunofluorescent staining of c-Fos

The obtained brain sections were washed three times with PBS and then hatched with PBS containing 1% TritonX-100 for 30 min, followed by hatching with PBS containing 10% goat serum for 2 h to block undefined proteins. The sections were hatched with primary antibody (c-Fos, 1:200 dilution, Abcam ab208942, RRID: AB_2313624), overnight at 4 °C. Subsequently, the sections were washed three times with PBS at room temperature, and hatched with Alexa fluor^®^594-conjugated goat anti-mouse IgG (1:400 dilution, Jackson ImmunoResearch AB_2338059) for 1 h at 37 °C and 1 h at room temperature. Finally, the imaging was completed using an Olympus VS120 Slide Scanner microscope.

### 2.6 Stereotaxic surgery

*Surgery* Mice were anesthetized with 1% pentobarbital sodium, and then secured in a stereotaxic apparatus (RWD Life Science, Shenzhen, China). The scalp was shaved, disinfected with povidone-iodine solution, and aseptically prepared. A midline sagittal incision (0.5**–**0.8 cm) was made using sterile surgical scissors to expose the skull. Superficial connective tissue overlying the skull was gently removed with sterile saline-moistened cotton swabs. The skull surface was leveled to establish the bregma as the zero point, ensuring horizontal alignment within a tolerance of < 0.02 mm. Craniotomies were drilled at target stereotaxic coordinates using a microdrill. Viral vectors were microinjected at a rate of 30 nL/min. Following injection completion, the microinjection needle remained *in situ* for 10 min to permit diffusion. The needle was subsequently withdrawn slowly. The incision was sutured and topical erythromycin ointment was applied to prevent infection. Mice received immediate subcutaneous administration of carprofen (5 mg/kg; MedChemExpress, China) for analgesia and anti-inflammatory effects for 3 consecutive days. Carprofen stock solutions were prepared in dimethylsulfoxide (DMSO, MedChemExpress, China) and diluted with 0.9% saline prior to subcutaneous administration in mice. The mice were placed on a heating pad to recover from anesthesia.

*Virus injection* Viral microinjections of rAAV-hSyn(Gq)-mCherry (50 nL/side, titer: 5.03 × 10¹² vg/ml; Braincase, China), rAAV-hSyn-hChR2(H134R)-EYFP (50 nL/side, titer: 5.03 × 10¹² vg/ml; Braincase, China), and rAAV-empty (50 nL/side, titer: 1.00 × 10¹² vg/ml; Braincase, China) were performed into the CeA (anteroposterior (AP) -1.65 mm, mediolateral (ML) ± 2.7 mm, dorsoventral (DV) -4.15 mm) (**Supplementary Figure S1, Supporting Information**). The coordinates used in this study were selected according to *The Mouse Brain in Stereotaxic Coordinates*, 4th edition (Paxinos & Franklin, 2013). Each mouse was injected with only one type of virus, with rAAV-empty serving as the negative control.

*Implantation fiber optic cannulas and microcatheter* For optogenetic experiments, customized fiber optic cannulas [Ø1.25-mm stainless ferrule, Ø200-mm core, 0.39 numericalaperture, 5.0 mm; Bogao Optoelectronic, Xi’an, China] was implanted to target the CeA (AP -1.65 mm, ML ±2.7 mm, DV -3.95 mm). 454 glue (Loctite, USA) and dental cement (Shanghai New Century Dental Materials Co., Ltd. China) were applied to secure the catheter. After the cement solidified, mice were placed on a heating pad for recovery from anesthesia, and postoperative care was provided. For Intracerebral drug administration experiment, using the same surgical procedure as for optical fiber implantation, a microcannula (outer diameter D = 26 G; injector protrusion length G1 = 0.5 mm; cannula cap core protrusion length G2 = 0 mm; metal tube length C = 5.0 mm; RWD Life Science, Shenzhen, China) was implanted into the CeA (AP -1.65 mm, ML ±2.7 mm, DV -3.95 mm), followed by postoperative care (**Supplementary Figure S2, Supporting Information**).

### 2.7 Measurement of food behavior

21 days after viral expression, mice were singly housed and fasted for 12 h. For chemogenetic experiments, mice expressing rAAV-hSyn(Gq)-mCherry or rAAV-empty received intraperitoneal injections of either 0.9% saline (Control group) or clozapine-N-oxide (CNO; 0.3 mg/kg; Sigma-Aldrich, St. Louis, MO, USA; prepared in DMSO and diluted with 0.9% saline). Standard chow was provided 1 h later, and food intake was measured at 1, 2, and 3 h after feeding. Feeding behavior during both light and dark cycles was assessed using the same protocol.

For open-field feeding tests, mice that were microinjected with a chemogenetic virus underwent the same fasting and injection procedures. One hour after injection, they were placed in an open-field arena (40 × 40 × 35 cm) divided into a 3 × 3 grid, with food pellets positioned in the corner zones. Locomotor activity and food acquisition were recorded using EthoVision XT software (Noldus, Netherlands).

In optogenetic experiments, mice received injections of rAAV-hSyn-hChR2(H134R)-EYFP or rAAV-empty. Following a 21-day post-surgery recovery period, the mice were tested using the same open-field paradigm as described above. Blue light stimulation (470 nm; Shanghai Fiblaser Technology, China) was delivered at 10 Hz with a 10 ms pulse width for 10 min during the test. Feeding behavior in the open field was monitored during optical stimulation.

### 2.8 Stereotaxic injection of GLP-1R agents

One week after recovery from microcannula implantation, mice were fasted for 3 h prior to the test. Liraglutide, Ex-4 (MedChemExpress, China), and exendin-9 (Ex-9; MedChemExpress, China) were dissolved in artificial cerebralspinal fluid (ACSF, Beijing Solarbio Science & Technology Co., Ltd, Beijing, China). Mice then received liraglutide (10 nmol), Ex-4 (0.05 µg), or Ex-9 (10 µg) via the implanted catheter. Control mice received equivalent volumes of ACSF. One hour after injection, standard chow or kaolin were provided, which were recorded at 1, 3, 6, and 24 h after provision, and body weight was measured before and after the test.

### 2.9 Data analysis

Data are presented as mean ± standard error of the mean (SEM), and statistical analyses were performed with GraphPad Prism software (RRID: SCR_002798). The analyses of behavior tests were accomplished by ANY-maze software (RRID: SCR_014289). Statistical analysis and comparison are generated by blind counting. The statistical significance of the differences between the groups was determined by Student’s *t* test, Two-tailed paired *t* test, and one-way analysis of variance (ANOVA). *p < 0.05, **p < 0.01, ***p < 0.001, and ****p < 0.0001.

## 3 Results

### 3.1 Administration of peripheral liraglutide reduced body weight and activated central brain regions

**Figure 1:**
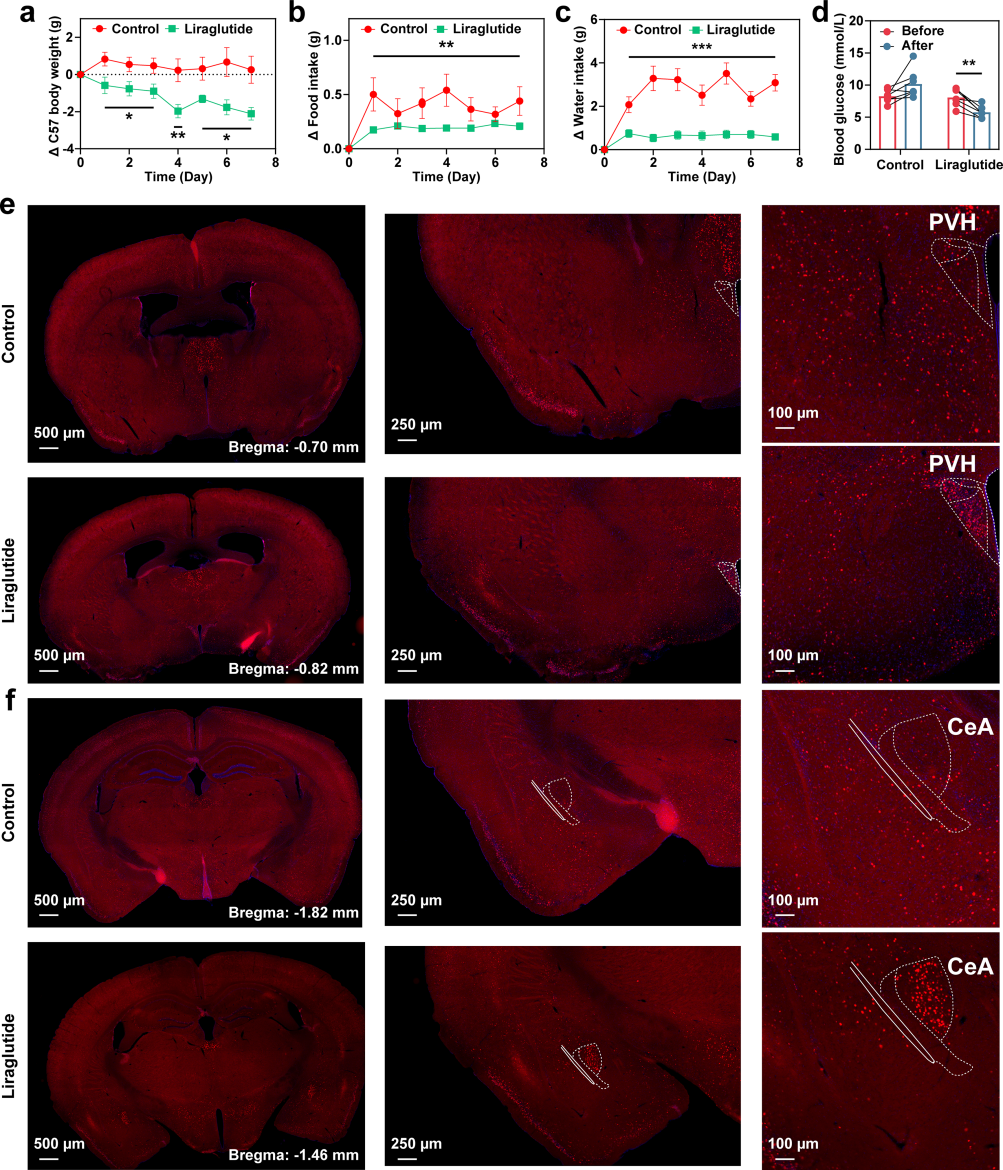
Administration of peripheral liraglutide regulated feeding. The change of body weight **(a)**, food intake **(b)**, and water intake **(c)** within a week under the condition of subcutaneous injections of liraglutide or saline (n = 7, Student’s *t* test). **(d)** The change of blood glucose before and after subcutaneous injections of liraglutide or saline (n = 7, Two-tailed paired *t* test). Representative images showing the significant differential expression of c-Fos in the central brain regions PVH **(e)** and CeA **(f)** after tail vein injection of liraglutide or saline (n = 3). All data were expressed as mean ± SEM, and asterisks indicate a significant difference (*p < 0.05, **p < 0.01, ***p < 0.001) as compared with the control group using Student’s *t* test and Two-tailed paired *t* test.

Liraglutide obviously reduced the mice body weight compared to the control group through subcutaneously injected into the neck (**Figure 1a**). Compared with the control group, the food (**Figure 1b**) and water (**Figure 1c**) intake of liraglutide group significantly decreased. Moreover, with the administration of liraglutide for a week, the level of blood glucose was reduced while it was not in the control group (**Figure 1d**). Remarkably, tail intravenous injection of liraglutide clearly activated the PVH and CeA (**Figures 1e, f**, **Supplementary Figure S3, Supporting Information**).

### 3.2 Gastric wall-injected PRV targets the central brain regions

**Figure 2:**
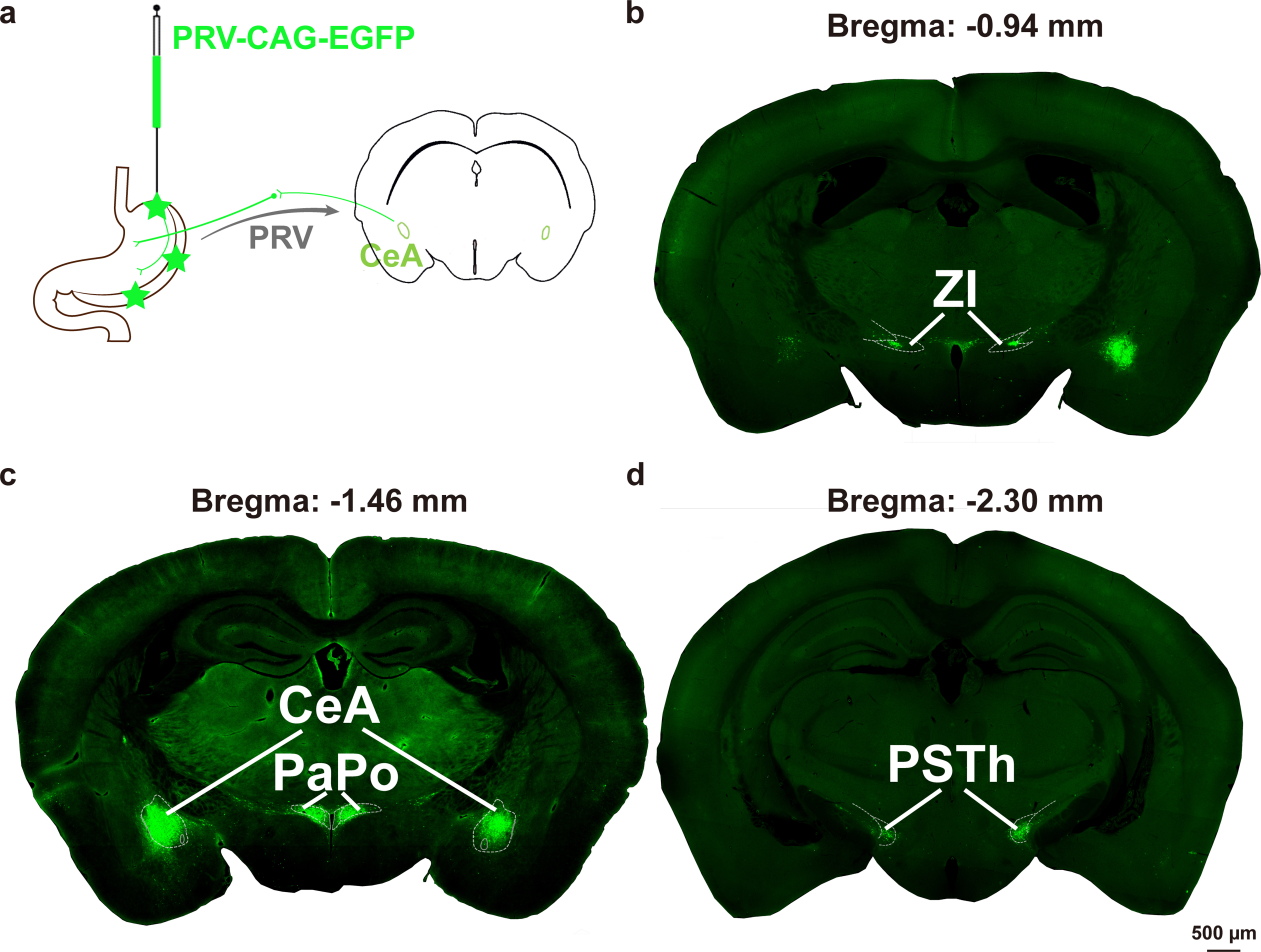
Neurotropic virus PRV was injected into the gastric wall to label the central brain regions. **(a)** Schematic of PRV-CAG-EGFP injection into the gastric wall. Whole-slide imaging showing PRV-infected brain regions of ZI **(b)**, CeA and PaPo **(c)**, and PSTh **(d)** (n = 3).

In order to clarify the central brain regions involved in the regulation of gastric function, the experiment used the neurotropic virus PRV for retrograde labeling (**Figure 2a**, **Supplementary Figure S4, Supporting Information**). Five days after PRV injection, several brain regions were infected, including the zona incerta (ZI) (**Figure 2b**), the CeA, the posterior paraventricular hypothalamic nucleus (PaPo) (**Figure 2c**), and the parasubthalamic nucleus (PSTh) (**Figure 2d**).

### 3.3 Activation of CeA through genetic methods inhibited mice feeding

**Figure 3:**
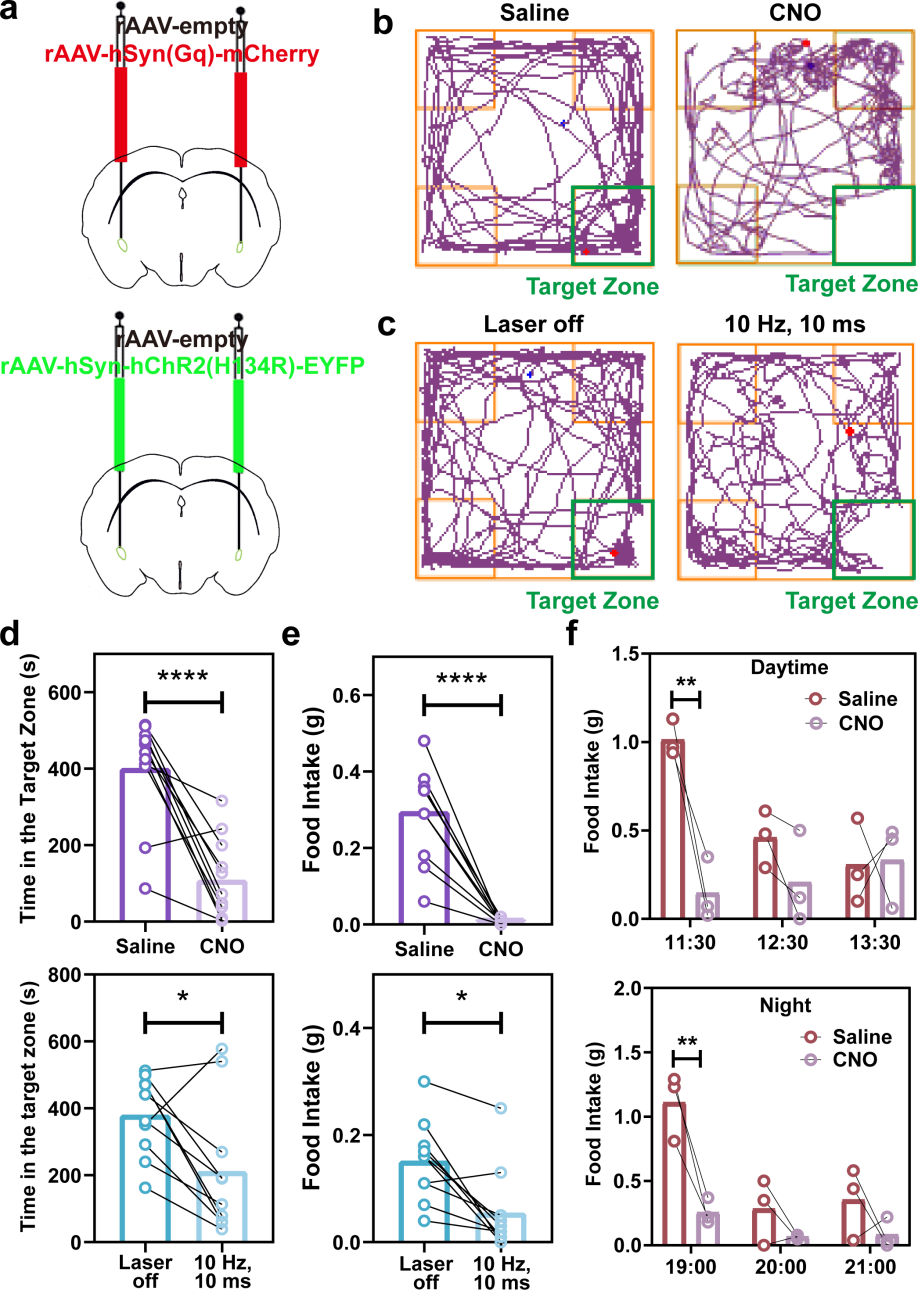
CeA activating by chemogenetics and optogenetics regulates mice feeding. **(a)** Schematic of stereotaxic injection of chemogenetic and optogenetic viruses. **(b)** Representative locomotor trajectories of mice 21 days after stereotaxic injection of rAAV-empty or rAAV-hSyn(Gq)-mCherry following intraperitoneal administration of saline or CNO. **(c)** Representative locomotor trajectories of mice 21 days after stereotaxic injection of rAAV-empty or rAAV-hSyn-hChR2(H134R)-EYFP following optogenetic activation via implanted optical fibers using 470-nm light at 10 Hz with a 10-ms pulse width. Time spent in the target zone **(d)** and food intake **(e)** before and after activating CeA (n = 8 or 9). **(f)** The food intake after chemogenetic activating CeA during 3 h on daytime and night (n = 3). All data were expressed as mean ± SEM, and asterisks indicate a significant difference (*p < 0.05, **p < 0.01, ****p < 0.0001) as compared with the saline/laser off group using Two-tailed paired *t* test.

The chemogenetic and optogenetic methods were employed to activate the CeA brain region in mice separately (**Figure 3a**) and observed their locomotor trajectories and feeding behavior in an open field (**Figures 3b, c**). The results showed that mice with an activated CeA spent significantly less time in the target zone where food was placed (**Figure 3d**), and their food intake also notably decreased (**Figure 3e**). Furthermore, when CNO was administered intraperitoneally to freely moving, singly housed mice to activate the CeA, food intake during the first hour after food presentation was significantly reduced compared with the saline-treated group (**Figure 3f**).

### 3.4 CeA GLP-1R agonists or antagonist microinjections manage food intake and nausea-like behaviors

**Figure 4:**
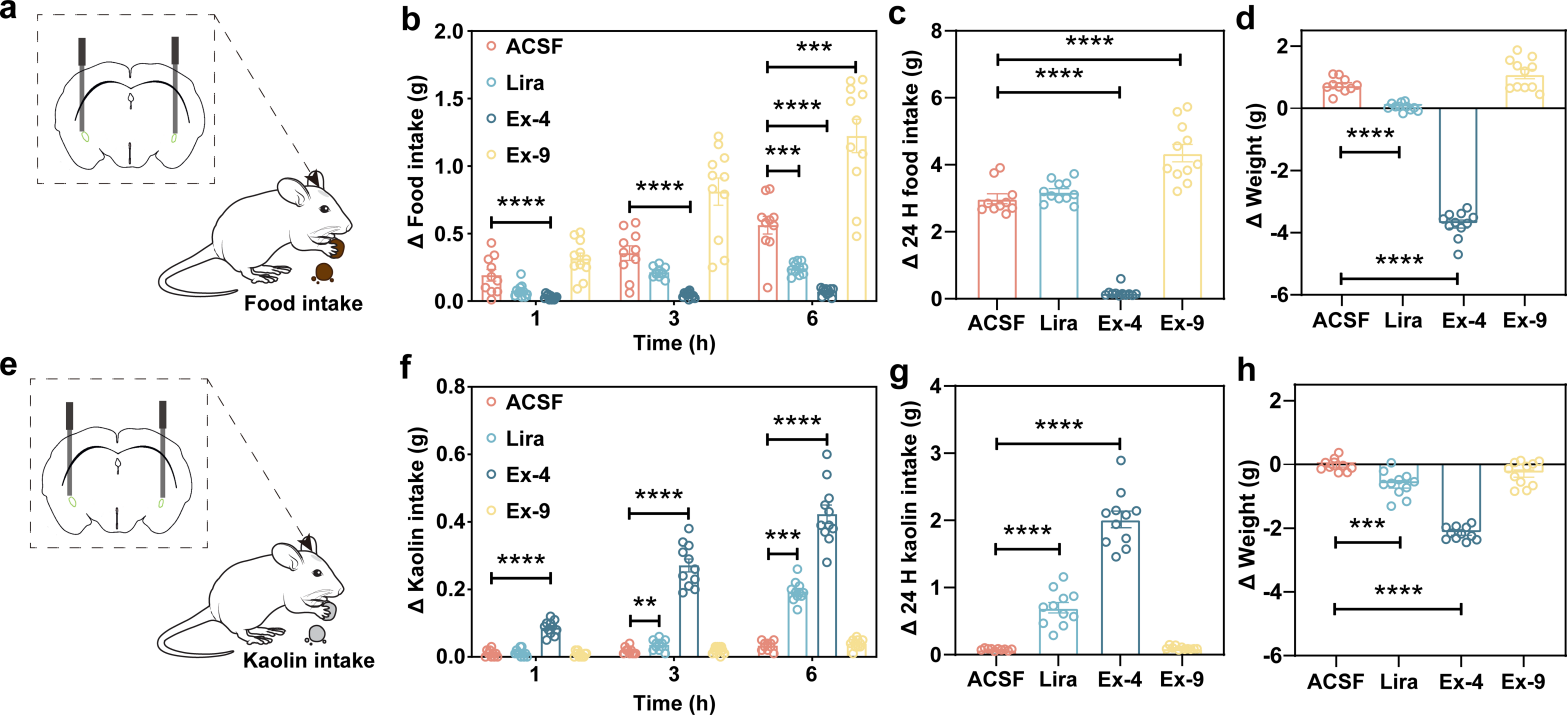
CeA regulates food and kaolin intake via GLP-1R agonists and antagonists. **(a)** Schematic of cannula-based drug administration and measurement of food intake in mice. **(b)** The food intake after microinjection of ACSF, liraglutide, Ex-4, and Ex-9 into CeA at 1 h, 3 h, and 6 h (n = 10 or 11). **(c)** The 24 h food intake after microinjection ACSF, liraglutide, Ex-4, and Ex-9 (n = 10 or 11). **(d)** The change of body weight during 24 h after microinjection ACSF, liraglutide, Ex-4, and Ex-9. **(e)** Schematic of cannula-based drug administration and measurement of kaolin intake in mice. **(f)** The kaolin intake after microinjection ACSF, liraglutide, Ex-4, and Ex-9 into CeA at 1 h, 3 h, and 6 h (n = 10 or 11). **(g)** The 24 h kaolin intake after microinjection ACSF, liraglutide, Ex-4, and Ex-9 (n = 10 or 11). **(h)** The change of body weight during 24 h after microinjection ACSF, liraglutide, Ex-4, and Ex-9 (n = 10 or 11). All data were expressed as mean ± SEM, and asterisks indicate a significant difference (**p < 0.01, ***p < 0.001, ****p < 0.0001) as compared with the ACSF group using the one-way ANOVA.

Administered GLP-1R agonists or antagonist to mice brains via indwelling catheters (**Figures 4a, e**) revealed that compared to the ACSF group, liraglutide significantly reduced mice feeding at 6 h after administration (**Figure 4b**), but the mice feeding returned to normal level at 24 h (**Figure 4c**). In contrast, Ex-4, a potent early-used GLP-1R agonist, significantly decreased mice feeding at 1 h after administration (**Figure 4b**) and continued to do so up to 24 h (**Figure 4c**). Ex-9, a GLP-1R antagonist, increased mice feeding significantly at 6 h after administration (**Figure 4b**) and still showed a stimulatory effect at 24 h (**Figure 4c**). After the 24 h recording ended, compared to the initial body weight of the mice, a significant decrease was detected in the liraglutide and Ex-4 groups (**Figure 4d**). Moreover, the results showed that compared to the ACSF group, after 3 h of liraglutide treatment, the kaolin intake of mice significantly increased (**Figure 4f**) and persisted until 24 h (**Figure 4g**). Surprisingly, one hour after the Ex-4 treatment, kaolin intake significantly increased (**Figure 4f**) and continued until 24 h (**Figure 4g**), suggesting that Ex-4 caused a more pronounced gastric dysfunction than liraglutide. However, there was no significant difference in kaolin intake compared to the ACSF group after Ex-9 treatment within 24 h (**Figures 4f, g**). The body weight of mice significantly decreased after the liraglutide and Ex-4 treatment compared to the ACSF group (**Figure 4h**).

## 4 Discussion

This present study showed that PRV injection into the gastric wall provides anatomical evidence that the CeA of male mice is involved in neural circuits regulating gastric function. Furthermore, site-specific GLP-1R agonist administration indicates that the CeA responds to GLP-1R activation to promote hypophagia and gastric reflex-related nausea-like behaviors, with liraglutide displaying a milder anorectic effect than Ex-4.

### 4.1 CeA involvement in gastric function regulation

Existing evidence suggests the presence of neural connectivity between the CeA and the DVC, which receives visceral inputs (25). Electroacupuncture stimulation mitigates restraint-stress-induced and irregular-feeding-induced gastric peristalsis through the CeA GABAergic neuron-DVC neuronal circuitry (18). Cell-specific ablation studies have demonstrated that GLP-1R^+^ neurons in the DVC play a crucial role in mediating anorexia induced by the peripheral administration of Ex-4 or semaglutide (8). These findings suggest that the CeA constitutes part of the central neural circuitry involved in the regulation of gastric function. This study reported that retrograde transsynaptic PRV injection into the gastric wall provide anatomical evidence supporting a connection between the stomach and the CeA. Moreover, stereotaxic administration of GLP-1R agonists into the CeA induced pronounced pica behavior in mice, which is commonly regarded as a behavioral correlate of anorexia and gastric malaise in rodents (26, 27). These results further support the involvement of the CeA in mediating gastric discomfort associated with GLP-1R agonist-induced hypophagia following site-specific administration.

### 4.2 CeA regulates feeding behavior through microinjection of GLP-1R agonists and antagonists

Previous rigorous whole-brain imaging studies have demonstrated that fluorophore-conjugated GLP-1R agonists preferentially accumulate in circumventricular organs, particularly the ARC and DVC (28, 29). Nevertheless, both peripheral and intracerebroventricular administration of GLP-1R agonists robustly induce Fos expression in the CeA (9), consistent with our observations. Importantly, this CeA Fos induction can be blocked by pretreatment with a GLP-1R antagonist (30), indicating that GLP-1R agonist-evoked Fos activation in the CeA requires GLP-1R signaling, despite the fact that c-Fos is not an ideal marker of neuronal activation (31). Furthermore, our results indicate that chemogenetic and optogenetic activation of the CeA influences feeding behavior in mice (**Figure 3**). Intracerebral administration of liraglutide or Ex-4 via an indwelling cannula markedly reduced food intake in mice, whereas Ex-9, a GLP-1R antagonist, produced the opposite effect (32) (**Figure 4**), which indicates that CeA mediates mice feeding behavior through intracerebroventricular administration of GLP-1R agonists and antagonists. Moreover, 24 h food intake monitoring revealed that microinjection of different GLP-1R agonists into the CeA produced distinct effects, with Ex-4 eliciting strong anorectic and pica behaviors, whereas liraglutide induced milder effects, and food intake returned to baseline within 24 h. More specifically, site-specific administration of liraglutide induced pronounced pica behavior at 3 h and a significant reduction in food intake at 6 h, while total food intake at 24 h did not differ significantly from the ACSF group, This suggests that following the cessation of liraglutide-induced anorexia, mice experienced a period of compensatory feeding, and the observed reduction in body weight likely reflects the energy expenditure during the initial anorectic phase (33).

Nevertheless, the present study has several limitations. First, our conclusions were not further validated using *Glp1r-cre* mice, which would provide critical genetic targeting evidence. Second, we did not directly assess changes in feeding behavior following peripheral liraglutide administration using neuron-specific manipulation of the CeA. Third, the specific subnuclei within the CeA that regulate liraglutide-induced anorexia and nausea-like behaviors have not been anatomically defined. The CeA is composed predominantly of GABAergic neurons, which can be further subdivided into populations expressing PKCδ, somatostatin, or tachykinin 2. Previous neuroanatomical study has shown that GLP-1R-expressing neurons in the CeA exhibit the highest degree of overlap with PKCδ^CeA^ neurons (12). However, it remains unclear whether GABA^CeA^ neurons in general, PKCδ^CeA^ neurons, or GLP-1R^CeA^ neurons specifically are responsible for mediating the feeding-suppressive effects of GLP-1RAs. Fourth, post-treatment energy metabolism in mice was not assessed. These mechanistic questions will be systematically addressed in future studies to delineate the precise neural circuits involved.

## 5 Conclusions

This study directly confirmed the anatomical connection between the CeA and the stomach through retrograde transsynaptic PRV injections into the gastric wall. Furthermore, stereotaxic microinjection experiments demonstrated that the CeA not only responds to site-specific GLP-1R agonist-induced hypophagia but also participates in gastric nausea-like behaviors. Although further studies employing cell-type-specific and projection-targeted approaches are needed to establish causal relationships, the present work underscores the substantive anatomical connection between the CeA and the stomach in male mice, as well as the influence of the CeA on GLP-1R agonist-induced hypophagia and pica behaviors.

## Data Availability

The raw data supporting the conclusions of this article will be made available by the authors, without undue reservation.
