# Supplementary material for: Anatomical evidence links the stomach to the central amygdala, a region responsive to local GLP-1R agonist induced feeding and nausea-like behaviors in male mice
Source: Front Endocrinol (Lausanne). 2026 Jan 28;17:1740052. doi: 10.3389/fendo.2026.1740052 (PMC12890699; doi:10.3389/fendo.2026.1740052)

Results

Figure S1. Determination of stereotaxic coordinates for the CeA. Representative coronal sections adapted from *The Mouse Brain in Stereotaxic Coordinates*, 4th edition (Paxinos & Franklin, 2013), spanning anteroposterior bregma coordinates from −1.22 to −1.94 mm. The CeA is located within this anteroposterior range and extends approximately 0.72 mm along the anteroposterior axis. The images illustrate the anterior (a) and posterior (b) boundaries of the CeA across coronal sections. To ensure accurate injections, we selected a central position within this range as the stereotaxic injection coordinate (anteroposterior [AP], −1.65 mm; mediolateral [ML], ±2.7 mm; dorsoventral [DV], −4.15 mm), at which a fluorescently labeled virus was injected. Immediately after the injection, mice were transcardially perfused with 20 mL of 0.9% saline followed by 20 mL of 4% paraformaldehyde, and coronal brain sections were obtained. Whole-slide images showed robust viral fluorescence across the anteroposterior range of −1.22 (c) to −1.94 mm (d), with ML and DV distributions consistent with those shown in panels (a) and (b), indicating that these coordinates reliably target the CeA for viral and drug delivery. Panels (c) and (d) show the CeA labeled by the fluorescent virus, as indicated by the white boxes.


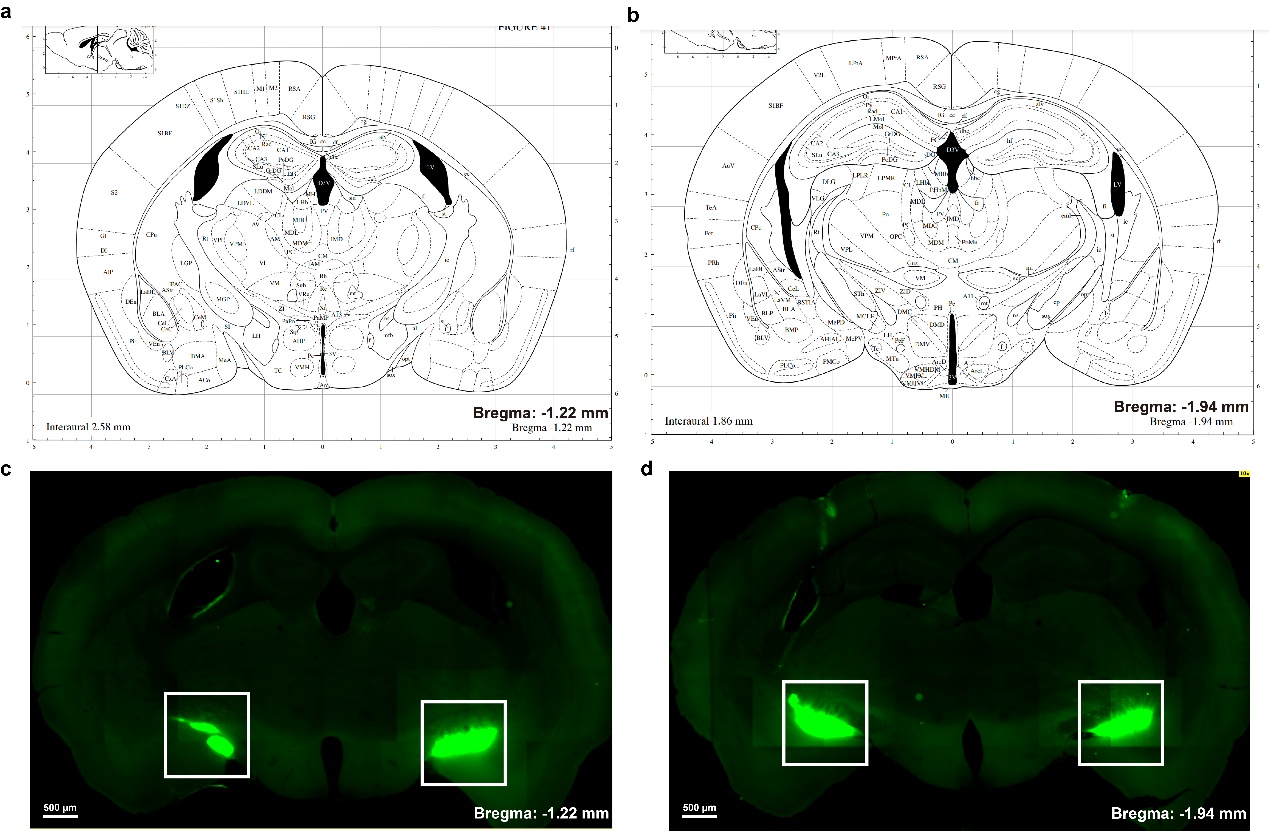


Figure S2. Representative images showing the relative location of the implanted microcannula within the brain. (a) Bright-field whole-slide coronal section, with the microcannula implantation site indicated by the black dashed box. (b) Hematoxylin and eosin–stained whole-slide coronal section, with the microcannula implantation site indicated by the black dashed box. Panels (a) and (b) depict the same brain region processed using different histological methods.


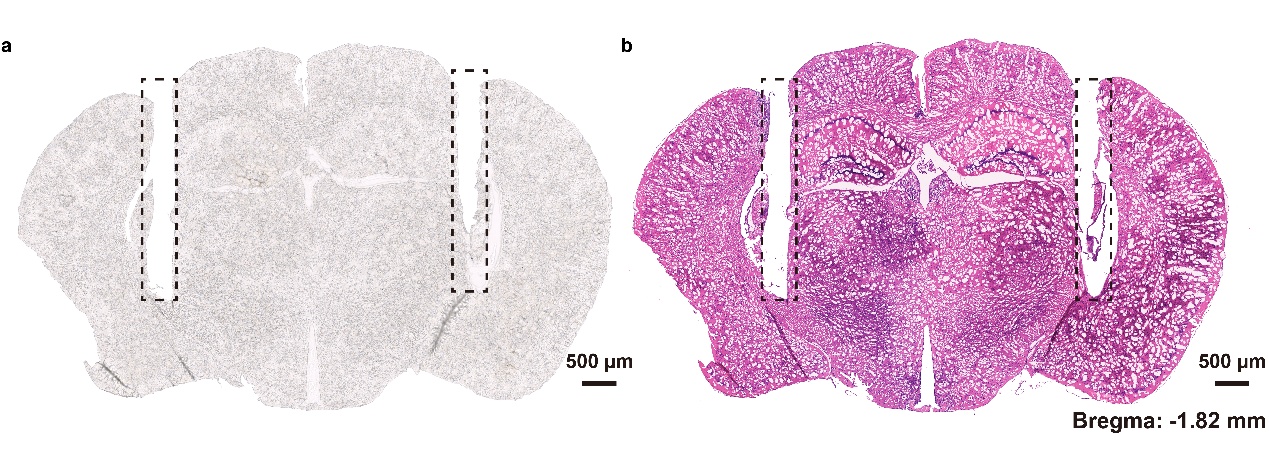


Figure S3. Coronal sections showing brain region activation following tail vein injection of liraglutide. (a) Activation of the PVH after tail vein injection of liraglutide. The control group received an equivalent volume of 0.9% saline (n = 3). (b) Activation of the CeA after tail vein injection of liraglutide. The control group received an equivalent volume of 0.9% saline (n = 3). The white boxes (red) indicate the approximate locations of the respective brain regions.


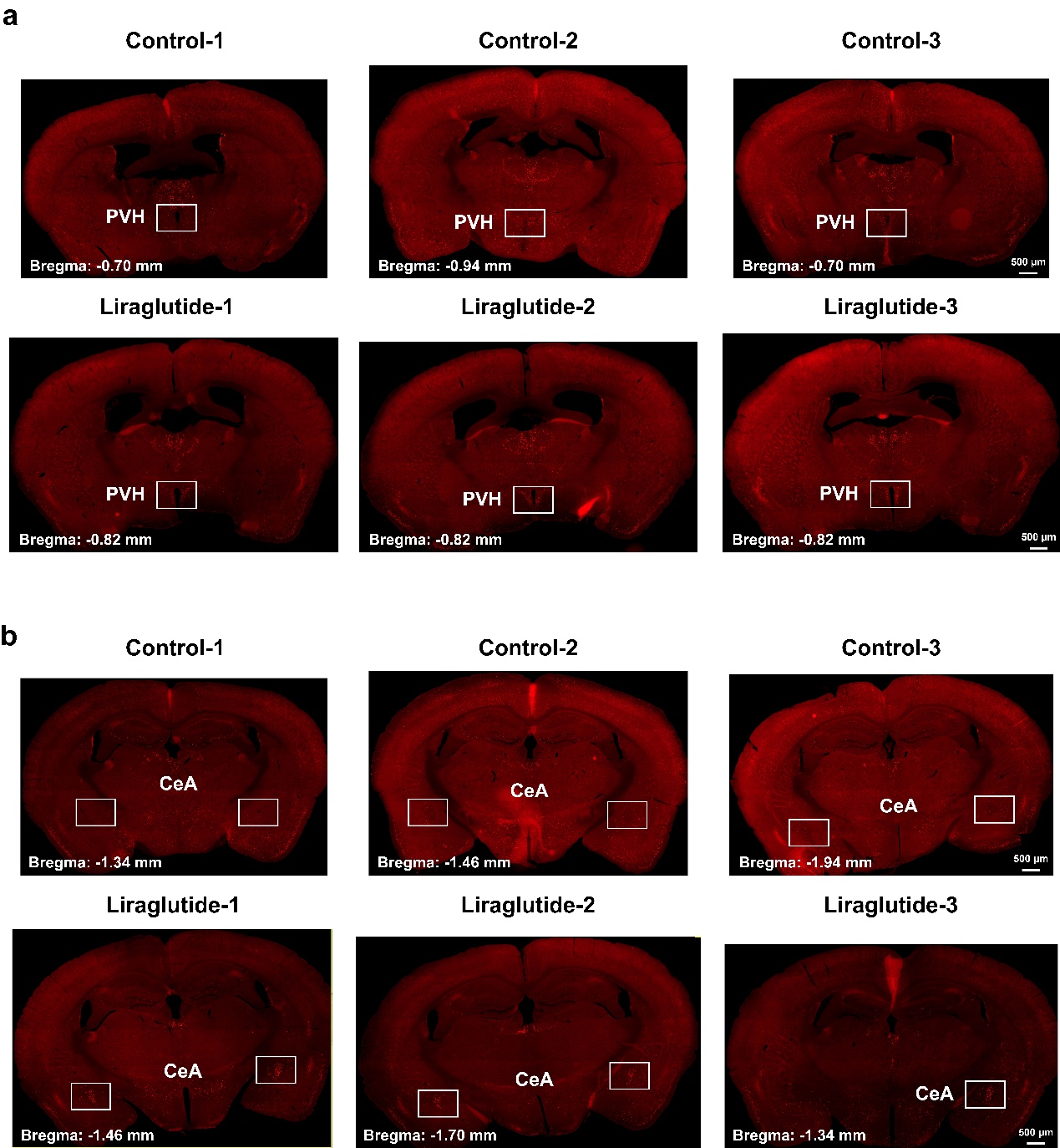


Figure S4. CeA infection in three mice following retrograde transsynaptic injection of PRV into the gastric wall (n = 3). The white boxes indicate the CeA (green) in each image.


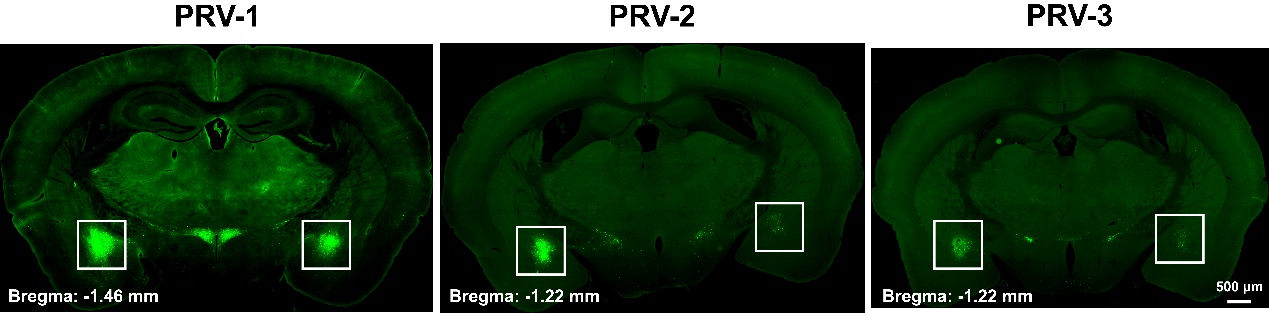

Supplement: Supplementary file 1 [file DataSheet1.docx]
